# Development and Validation of an Educational Tool on Hypodermoclysis for Palliative Care Professionals

**DOI:** 10.3390/nursrep15080301

**Published:** 2025-08-16

**Authors:** Maria Vanessa Tomé Bandeira de Sousa, Carlos Laranjeira, José Mateus Pires, Isabela Melo Bonfim, Luís Carlos Carvalho Graça, Karla Maria Carneiro Rolim, Lara Anisia Menezes Bonates, Régia Christina Moura Barbosa Castro, Ana Fátima Carvalho Fernandes

**Affiliations:** 1 Walter Cantídio University Hospital, Federal University of Ceará, Fortaleza 60430-372, CE, Brazil; mariatome.huwc@ebserh.gov.br; 2 School of Health Sciences, Polytechnic University of Leiria, Campus 2, Morro do Lena, Alto do Vieiro, Apartado 4137, 2411-901 Leiria, Portugal; 3 Centre for Innovative Care and Health Technology (ciTechCare), Polytechnic University of Leiria, Campus 5, Rua das Olhalvas, 2414-016 Leiria, Portugal; 4 Comprehensive Health Research Centre (CHRC), University of Évora, 7000-801 Évora, Portugal; 5 Department of Nursing, Federal University of Ceará, Fortaleza 60430-160, CE, Brazil; matheusp2010@gmail.com (J.M.P.); regiabarbcastro@gmail.com (R.C.M.B.C.); afcana@ufc.br (A.F.C.F.); 6 Department of Nursing, University of Fortaleza, Fortaleza 60811-905, CE, Brazil; isabelambonfim@hotmail.com (I.M.B.); karlarolim@unifor.br (K.M.C.R.); 7 Health Sciences Research Unit: Nursing (UICISA: E), School of Health, Polytechnic Institute of Viana do Castelo, 4900-347 Viana do Castelo, Portugal; luisgraca@ess.ipvc.pt; 8 Center of Health Sciences, State University of Ceará, Fortaleza 60714-903, CE, Brazil; larabonates1@gmail.com

**Keywords:** hypodermoclysis, palliative care, educational tool, validation studies, nursing

## Abstract

**Background/Objectives**: Hypodermoclysis has gained increasing recognition as a safe, effective, and minimally invasive method for administering medication and fluids in palliative care. Despite its advantages, its adoption remains limited, primarily due to a lack of structured training resources for healthcare professionals. This study aimed to develop and validate an educational tool for training clinical nurses in hypodermoclysis administration in palliative care. **Methods**: This is a methodological study with a multi-methods approach. Study development involved a needs assessment with 48 professionals, a literature review, and the creation of a manual enriched with visual aids. **Results**: The material was validated by expert judges, technical reviewers, and the target audience. Organized into 21 chapters, the manual comprehensively addresses technical, theoretical, and ethical dimensions of the practice. Content validation by 14 experts yielded an outstanding global Content Validity Index (CVI) of 0.95. An independent evaluation of visual design by four communication specialists produced consistently high scores (91–96%), classifying the material as “superior” in quality. Feedback from target users (12 nurses) highlighted the manual’s clarity, applicability, and relevance. All constructive suggestions were incorporated into the final version. **Conclusions**: The resulting manual demonstrates strong validity as an educational resource, with significant potential to standardize and enhance hypodermoclysis training in palliative nursing, promoting both safety and humanized care.

## 1. Introduction

Hypodermoclysis, also known as subcutaneous infusion, involves the administration of fluids and medication into the subcutaneous tissue. This technique has gained prominence in palliative care (PC) due to its safety, effectiveness, and ease of application, especially for patients with reduced oral intake or limited venous access. Despite these benefits, its adoption remains limited, mainly because of the lack of structured training resources for healthcare professionals [1]. Introduced in the 19th century, hypodermoclysis has long been recognized as a viable alternative in prolonged care settings, contributing meaningfully to patient comfort and dignity [2,3,4].

In the context of PC, which prioritizes symptom relief and quality of life, hypodermoclysis has become an essential practice for symptom management [2,3,4,5]. Its increasing utilization reflects the need to optimize care by reducing complications and promoting a more humanized approach, particularly for patients with limited prognosis [6].

Nevertheless, despite its effectiveness and safety, hypodermoclysis remains underutilized in PC, primarily due to a lack of standardization and insufficient technical knowledge among healthcare professionals [7,8]. The absence of validated protocols, educational materials, and clear guidelines regarding medication selection, dilution, and infusion timing creates uncertainty in clinical practice [9]. This scenario hampers nursing team adherence, limits the use of less invasive therapeutic options, and complicates the development of standardized protocols across diverse care settings [7,8].

Therefore, developing educational resources to standardize and enhance hypodermoclysis practice has become essential [10]. Evidence-based educational tools have proven effective in reinforcing best practices, improving the quality of care and promoting patient safety [11].

In this context, the creation and validation of educational materials designed for nurses providing care to patients in palliative settings represent a relevant strategy for improving the quality and safety of clinical practice. Consequently, this initiative seeks to address gaps in professional training by disseminating consistent, safe, and evidence-based practices [10]. This study aims to develop and validate an educational tool for training clinical nurses in hypodermoclysis administration in PC.

## 2. Materials and Methods

### 2.1. Study Design and Development Stages of the Educational Manual

**Figure 1:**
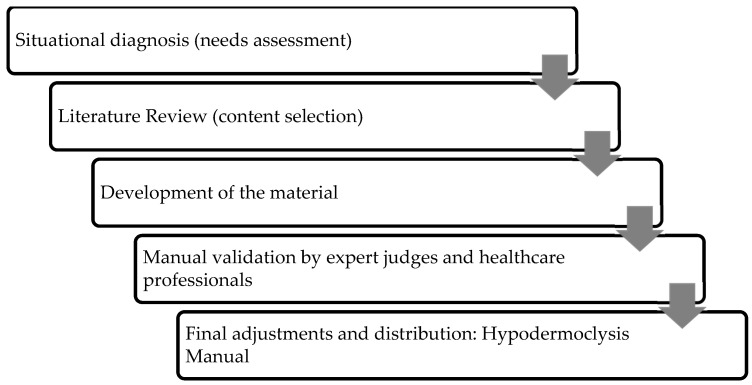
Stages of development and validation of the educational manual.

This methodological study with a multi-methods approach developed and validated a hypodermoclysis manual for clinical nurses [12]. The development and refinement of the educational material followed several stages (Figure 1): (a) situational diagnosis through a questionnaire applied to the target audience; (b) content selection based on a literature review; (c) development of the material: text composition, integration of illustrations, and pilot version production; (d) validation by experts and the target audience; and (e) final adjustments for layout, printing, and distribution [12].

#### 2.1.1. Situational Diagnosis

Between August and September 2023, a situational diagnosis was conducted with the target audience using a self-developed instrument divided into two parts: sociodemographic characterization and open-ended questions focused on the practice of hypodermoclysis in nursing. This stage aimed to guide the selection of content for the educational material. Convenience sampling was used, including nurses working with PC patients and with potential involvement in hypodermoclysis administration. Professionals on leave (vacation or medical leave) and those who did not complete the questionnaire were excluded. Participation was voluntary, following the signing of the informed consent form and completion of the questionnaire, which was made available both online and in person.

As part of this phase, nurses’ perceptions were explored through three open-ended questions addressing: (1) essential knowledge about hypodermoclysis; (2) perceived difficulties and facilitators in performing the procedure; and (3) suggestions for content to be included in an educational manual. The questions were developed by the research team, composed of nurses with clinical expertise in palliative care and based on prior observations of knowledge gaps in routine practice.

The data obtained from the needs assessment were analyzed using thematic-categorical content analysis, supported by the software Interface de R pour les Analyses Multidimensionnelles de Textes et de Questionnaires (IRAMuTeQ; version 0.7 alpha 2) for textual processing [13,14]. This procedure enabled the identification of categories through Descending Hierarchical Classification (DHC), resulting from the processing of interviews regarding nurses’ experiences and needs related to the hypodermoclysis technique [14]. The information obtained served as the foundation for the manual’s construction, making it more appealing and accessible to the target audience.

#### 2.1.2. Literature Review

To support the development of the pilot project for the manual, an integrative literature review was conducted to identify evidence-based practices for the management of hypodermoclysis in PC. Searches were performed in April 2023 on the PubMed/MEDLINE, LILACS, Cochrane Library, and CINAHL databases, using controlled DeCS/MeSH descriptors: “Palliative Care,” “Hypodermoclysis,” “Subcutaneous Infusions,” “Nursing Care,” and “Patient Safety”. Synonymous terms within each concept were combined with the Boolean operator OR, and the main concepts were intersected using AND. No time frame was established.

Primary studies, both qualitative and quantitative, as well as secondary studies that addressed the hypodermoclysis technique, its applicability, and safety within PC contexts, were included. Editorials, letters to the editor, conference abstracts, and monographs were excluded; only full-text publications were considered. The selection process followed the stages of identification, screening, eligibility, and inclusion. Extracted data were analyzed through critical reading and narrative synthesis, considering methodological aspects and the main findings related to the effectiveness, safety, and applicability of the technique.

Extracted data were analyzed through critical reading and narrative synthesis, considering methodological elements such as study design, population characteristics, type of intervention, measured outcomes, data collection and analysis procedures. A structured appraisal instrument, developed by the authors, was used for this purpose. The process was conducted independently by two reviewers to ensure methodological consistency and analytical rigor.

#### 2.1.3. Development of the Material

The content of the educational manual was developed based on the findings of the literature review and the needs identified in the situational diagnosis conducted with nurses, ensuring conceptual clarity, scientific grounding, and practical safety. The preliminary version was drafted using Microsoft Word^®^ 2016 and contained the initial textual framework of the educational tool.

Following the textual development, the material was submitted for layout design by a professional graphic designer, with illustrations to ensure an attractive and easily understandable presentation. At this stage, adjustments were made to the content, layout, and design, which were discussed during team meetings in order to produce a refined version for the validation phase.

#### 2.1.4. Content and Design Validation

Subsequently, the manual underwent content and visual design validation, involving expert judges in the fields of PC, pharmacy, patient safety, and graphic design, in addition to representatives of the target audience. The selection of expert judges followed Fehring’s adapted criteria [15], chosen to ensure greater accuracy and reliability in the evaluation process. These criteria, originally developed for the validation of nursing diagnoses, considered academic qualifications, professional experience, and expertise in the subject matter. Potential candidates were identified through professional networks and a search and review of academic curricula, after which invitations were sent individually via email. A convenience sampling approach was employed for the final selection.

The metric used for content validation was the Content Validity Index (CVI), which measures the degree of agreement among experts on a given topic. The instrument applied to the content judges was structured in two parts. The first concerned the identification of the evaluators, while the second consisted of items organized into the domains of objectives, structure and presentation, and relevance of the manual [16]. The objectives domain addressed the purposes and intended goals of the educational material. The structure and presentation domain referred to the organization of the guidelines, including general structure, presentation strategy, coherence, and formatting. The relevance domain evaluated the significance and importance of the content presented in the manual. This structure ensured a detailed analysis of the manual’s effectiveness in achieving its educational purpose.

Each item in the instrument was rated using a four-point Likert scale: 1 for items deemed not relevant or unclear, 2 for those requiring substantial revision, 3 for items requiring minor adjustments, and 4 for items considered relevant and clear. Scoring was based on the sum of responses rated 3 or 4, while items rated 1 or 2 were subject to revision. Items were considered valid if they achieved a CVI of 0.80 or higher [17]. To ensure anonymity, evaluators were identified by the letter “JC” followed by a sequential number (e.g., JC1, JC2, JC3).

For the evaluation of the design by technical judges, the material’s feasibility for its intended purpose was assessed using an adapted version of the Suitability Assessment of Materials (SAM) instrument. This tool classifies materials according to the following percentages: 70–100% as “Superior Material,” 40–69% as “Adequate Material,” and 0–39% as “Inadequate Material” [18]. It consists of 22 items organized into six dimensions: content, literacy demand, graphics, layout and typography, motivation, and cultural appropriateness. Each item is rated on a three-point scale (0 = inadequate, 1 = adequate, 2 = superior), and the total score was converted into a percentage to determine the overall material classification. Eight professionals were invited to participate in this phase, using a snowball sampling technique; however, only four accepted.

The adequacy score was calculated based on criteria such as content, language, presentation, illustrations, motivation, and cultural appropriateness. Each criterion was rated according to its level of adequacy: 2 points for “Superior,” 1 point for “Adequate,” and 0 points for “Inadequate.” The final score was obtained by summing all assigned points, dividing by the maximum possible score (number of criteria multiplied by 2), and multiplying the result by 100 [18]. To maintain anonymity, judges were identified by the abbreviation “JD” followed by a number (e.g., JD1, JD2, JD3).

Following the content and visual design validation, the manual was also submitted to evaluation by the target audience, composed of 12 nurses working in PC. Data was collected using an instrument developed by the research team, containing open-ended questions designed to assess aspects such as clarity, applicability, relevance, language, illustrations, and cultural appropriateness of the material. The responses were submitted to a structured content analysis process, which involved initial organization of the material, identification of relevant themes, and categorization of recurring elements [13]. The analysis identified patterns related to clarity, applicability, relevance, language, visual aspects, organization and logical sequence of information, as well as cultural and motivational suitability for clinical use.

#### 2.1.5. Final Adjustments and Distribution

After the validation stages involving experts and the target audience, the received feedback was incorporated into the manual to improve its usability and alignment with clinical practice in PC. Subsequently, a final revision phase was conducted, including textual review to correct any typographical errors and to enhance the clarity and objectivity of the content. The final version was formatted as a PDF (Portable Document Format) file, optimized for printing on A4 paper, in accordance with readability and legibility criteria. A professional graphic designer also made visual adjustments, such as repositioning images and modifying the spacing between text and illustrations, resulting in an attractive and functional layout. The manual is currently seeking institutional approval for future release and distribution within the hospital network, aiming for broad use by nursing professionals involved in PC.

## 3. Results

### 3.1. Situational Diagnosis and Manual Development

The development of the manual was grounded in a situational diagnosis of the target audience, which identified knowledge gaps and defined priority topics. A total of 48 healthcare professionals participated in this stage, of whom 44 (91.5%) were nurses, the majority being female. Regarding place of origin, 42 (87.2%) were from Lisbon, the capital of Portugal. The predominant age group was 40 to 49 years (12 participants, 25%), followed by 30 to 39 years (11 participants, 22.9%) and 50 years or older (8 participants, 16.7%). In terms of educational background, 24 participants (50%) held a master’s degree, 16 (33.3%) were specialists, 6 (12.5%) held doctoral degrees, and 2 (4.2%) had undergraduate degrees. As for professional experience, most participants had between 6 and 10 years of practice (40%), followed by those with up to 5 years (19%). The remainder had more than 10 years of experience (41%).

The open-ended questions from the situational diagnosis questionnaire helped identify knowledge gaps and topics deemed essential by nurses for the safe and effective use of hypodermoclysis. Content analysis of the responses highlighted the need to address topics in the manual related to clinical practice, technical care, and complication management. These findings underscored the importance of a didactic, objective, and context-appropriate educational resource.

Concurrently, the integrative literature review provided updated scientific evidence on the use of hypodermoclysis in PC. The search initially retrieved 68 records from electronic databases. After removing two duplicates and screening titles and abstracts, 58 records were excluded for not meeting the inclusion criteria. Eight studies were finally included in the review, comprising randomized controlled trials, descriptive observational studies, and clinical protocols. The synthesis of selected articles revealed practical recommendations, factors associated with adverse events, and training experiences, confirming and expanding upon the findings from the situational diagnosis. The content extracted from the literature was incorporated into the manual.

Based on the data collected, the informational needs were analyzed to support the development of an educational tool with accessible language tailored to the target audience. As a result, the first version of the “Hypodermoclysis Manual” was created, structured into 21 chapters covering topics from an introduction to PC (history, meaning, and principles) to symptom control, patient safety, skin and lymphatic system aspects, as well as technical guidelines for administering hypodermoclysis.

The manual also included content on regulatory guidelines, advantages and disadvantages of the technique, fundamentals of pharmacokinetics and bioavailability, recommended and contraindicated sites of administration, complications and adverse events, medication administration, off-label use, incompatible drugs, technique execution, nursing care, standard operating procedures (SOP), and post-puncture care.

Following formatting and revision, the first version of the manual was finalized with a total of 58 pages. The document was structured in Portable Document Format (PDF), optimized for printing on A4 paper (297 × 210 mm), and submitted for expert review as part of the educational material refinement process. This stage is essential to ensure that the manual meets criteria of relevance, clarity, and practical applicability, thus enhancing nurses’ competencies in using hypodermoclysis as a tool in PC settings.

### 3.2. Content Validation

The validation of the Hypodermoclysis Manual was conducted in two complementary stages: content validation and visual design validation. This process followed rigorous analytical criteria to ensure the technical, scientific, and visual quality of the material.

The content validation stage involved 14 expert judges, of whom 13 (92.9%) were nurses and 1 (7.1%) was a pharmacist. Regarding academic background, one judge (7.1%) held a postdoctoral degree, eight (57.1%) had master’s degrees, and five (35.7%) were specialists. Ten judges (71.4%) reported having practical experience with the subject matter, while 7 (50%) had previously published scientific papers on the topic. Twelve judges (85.7%) indicated prior experience with the validation of educational tools, and nine (64.3%) reported teaching experience.

The validation was organized into three main categories, as defined by the evaluation instrument: objectives, structure and presentation, and relevance. For each item, the Content Validity Index (CVI) was calculated, as detailed in Table 1.

All comments related to the content’s structure and organization referred to item 2.4 (Structure/Presentation). Two evaluators considered the logical sequence to be partially adequate, while one judge recommended improvements to the overall organization. These suggestions were incorporated into the revised version of the manual. In the “structure and presentation” category, a satisfactory average CVI was achieved; one item (also related to content organization) was rated as inadequate by a judge. In the ‘Relevance’ category, all items achieved a CVI of 1.00, reflecting full agreement among experts. However, two items in the overall instrument received scores slightly below 1.00, resulting in a global CVI of 0.95.

In addition, the open-ended feedback provided by the expert judges contributed significantly to improving the manual, resulting in an educational resource that is clearer, more accurate, and better aligned with the needs of the target audience. These reflections are presented in Table 2, which outlines the specific recommendations, and the corresponding decisions made during the revision process.

The feedback was integrated into the manual, enhancing both its content and visual presentation. The revisions aligned the material with the target audience’s expectations and with the best educational practices.

### 3.3. Visual Design Validation

The visual design validation of the manual was carried out by four judges with experience in the fields of teaching, digital media, communication, and graphic design. The material received high scores in all evaluated categories, with average ratings ranging from 91% to 96%, indicating a positive assessment of both visual adequacy and content (Table 3). The “illustrations” category received the lowest score, with 75%, suggesting the need for minor adjustments. The manual was considered appropriate and met the criteria for classification as “Superior.” The suggestions received, such as correcting the positioning of images on certain pages, were implemented in the final version of the material.

All evaluated items classified the material as “superior,” with scores ranging from 75% to 96%. The lowest score was recorded in the “illustrations” item, with 12 points (75%). The “presentation” category received 14 points (88%), and “stimulation and motivation” totaled 21 points (88%). The remaining items received scores above 90%.

In the subjective evaluation by the visual design judges, additional observations were recorded to complement the quantitative data. Judge JD1 commented on the “language” item, suggesting that “some topics might benefit from visual elements that create textual hierarchy.” In the same item, JD3 noted that “the language is clear and accessible.”

Judges JD1, JD2, and JD4 highlighted the need to pay attention to spacing between text and illustrations. However, they awarded maximum scores to all items and praised the ease of understanding, considering the material to be clear and concise.

JD4 also remarked about the text’s length, stating: “I understand that, being a manual, it cannot be shorter, but the text does become lengthy. One suggestion would be to use a greater number of more attractive figures, such as tables, mind maps, or timelines.”

After analyzing the evaluations from the content and visual design judges, the manual was returned to the designer for corrections and adjustments. The updated version was then submitted for validation by the target audience.

### 3.4. Target Audience Validation

In this stage, the validation of the manual involved 12 clinical nurses out of a total of 25 invited nurses, resulting in a response rate of 48%. Most participants were female (n = 11; 91.7%). All professionals had more than 10 years of education and experience in clinical practice (n = 12; 100%). Regarding the age group, five participants (41.7%) were between 30 and 39 years old, four (33.3%) between 40 and 49, two (16.7%) between 50 and 59, and one (8.3%) between 60 and 69.

In the qualitative assessment, participants highlighted positive aspects related to the clarity, organization, and applicability of the manual. Recorded comments included: “Content-rich material that will guide safe practice for hypodermoclysis administration” (E3); “Comprehensive content, appropriate and simple language. Illustrations complemented the text in a didactic way” (E5); “Relevant content for direct care […], dynamic approach […], appropriate language. Images […] complement the text” (E6); and “Very illustrative material, with all the information […] relevant to the subject. It covers everything we need to know” (E11).

Participants positively evaluated the logical sequence of topics, the attractiveness of the cover, font size and type, and the adequacy of the illustrations. The text was described as clear, with accessible vocabulary and a smooth reading flow. It was also noted that the manual sparked interest and facilitated learning.

A suggestion was made to revise the placement of references in certain sections, especially when they appeared directly below titles, which made it difficult to identify the source of the information (E10). These suggestions were incorporated into the final version of the material.

The final version of the Hypodermoclysis Manual was structured based on the data collected throughout the study, including the results of the literature review, the situational diagnosis, the evaluations by expert judges, and the validation with the target audience. Supplementary Table S1 presents the manual’s table of contents with each section and its main topics.

## 4. Discussion

The development and validation of instructional materials are effective strategies for bridging training gaps in healthcare, particularly for underexplored topics such as subcutaneous infusion. In this context, evidence-based educational tools grounded in active learning methodologies promote long-term knowledge retention and improve clinical practice [11]. Materials developed with clear language, validated content, and appropriate visual presentation enhance the learning process and positively influence professional behavior.

Although hypodermoclysis is widely recognized as a safe and effective technique for administering medications and fluids, its use remains below its potential, primarily due to institutional barriers and a lack of professional training [19,20]. The absence of clear policies, standardized protocols, and specific training is believed to be one of the main obstacles in clinical settings. Validated educational materials can be essential for overcoming these barriers, promoting adherence and safe implementation of the technique [21].

The methodological approach adopted in this study aligns with recommendations that emphasize the active involvement of end users in the creation of educational materials [22]. This participatory approach, by directly integrating the perspective of healthcare professionals into content development, enhances the relevance and applicability of the materials produced, particularly in complex care settings such as PC. Moreover, the emphasis on robust scientific evidence and the use of appropriate instructional strategies strengthens the educational quality of the material, increasing its potential impact on clinical practice [22,23].

Another important methodological strength was the structured, multi-stage validation process, which involved both expert judges and representatives of the target audience. This approach supports rigorous evaluation and ensures that the material is assessed in terms of clarity, relevance, practical applicability, and visual adequacy, thereby promoting its acceptance and effectiveness in care settings [24].

Hypodermoclysis aligns with the principles of PC by providing safe symptom relief, even for frail patients. Its application, however, requires institutional support and individualized monitoring, particularly in cases of low functional performance [5]. In this scenario, the manual developed in this study is a strategic resource, offering guidance that can strengthen the autonomy and confidence of professionals and may even serve as a tool for training students [25].

Visual design is another essential component in the effective construction of educational materials, as it directly influences the efficiency of communicating complex information [26]. A clear and hierarchically organized visual presentation, as found in the developed educational tool, significantly contributes to reducing cognitive overload and facilitates the retention of technical content [25].

It is well established that well-structured and targeted materials improve decision making, reduce care-related risks, and promote adherence to best clinical practices [21]. This underscores the importance of ensuring that such resources are validated and aligned with the real demands of care, in order to maximize their positive impact on clinical practice [21]. In the present study, the manual achieved excellent scores in both content and appearance evaluations, and was widely approved by the target audience, indicating the methodological and pedagogical robustness of the developed tool.

It should be noted, however, that the effectiveness of these resources depends on their practical applicability in daily professional routines. The success of educational tools requires that content, language, and objectives be carefully aligned with the specific context of use [27]. The theoretical, technical, and regulatory integration present in the manual meets these principles, offering a solid and practical tool for day-to-day care and ensuring quality of treatment for all PC patients [28].

Although this study provides a significant contribution to the field, this study has certain methodological constraints that should be considered when interpreting the findings. The needs assessment relied on a convenience sample, and the design evaluation included a small number of experts, which may have influenced the diversity of perspectives. Participation rates among invited judges and target audience professionals were modest, which could have affected the breadth of contributions to content and design validation. As the study was conducted within a single institutional context, the transferability of the findings may be context-dependent. Despite this, the multi-method approach, active involvement of end users, and structured validation framework strengthen the methodological rigor and practical applicability of the educational tool. Furthermore, the validation indices obtained, particularly in terms of content clarity, relevance, and applicability, were consistently high, supporting the robustness of the findings. Future studies should expand the sample and longitudinally evaluate the effects of the manual on routine care, including adherence to the technique, patient safety, and quality of care.

## 5. Conclusions

This study developed and validated an educational manual on hypodermoclysis, with the potential to contribute to clinical practice within PC settings. The material provides standardized guidelines on a safe, effective, and feasible therapy, compiling up-to-date content on indication, preparation, administration, and monitoring. By strengthening nurses’ technical autonomy and reducing care-related risks, this tool addresses the lack of educational materials focused on subcutaneous interventions, promoting patient care centered on comfort, dignity, and safety. The positive validation by specialists, design professionals, and the target audience confirmed its clarity, applicability, and relevance, reinforcing its potential for use in diverse healthcare settings.

## Figures and Tables

**Table 1:** Evaluation by content and visual design judges regarding objectives, structure and presentation, and relevance.

Objective	CVI
1.1 Correspond to the educational needs concerning hypodermoclysis	1.000
1.2 Feasible for promoting behavioral and attitudinal changes among professionals	0.850
1.3 Suitable for dissemination within the scientific community of PC	1.000
**Structure/Presentation**	
2.1 The educational material is appropriate for guiding professionals who care for patients receiving PC	1.000
2.2 The messages are presented in a clear and objective manner	1.000
2.3 The information presented is scientifically accurate	1.000
2.4 The proposed content follows a logical sequence	0.920
2.5 The material is appropriate for the sociocultural level of the proposed target audience	1.000
2.6 The information is well-structured with proper grammar and spelling	1.000
2.7 The writing style matches the knowledge level of the target audience	1.000
2.8 The information on the cover, back cover, acknowledgments, and/or foreword is coherent	1.000
2.9 The illustrations are expressive and sufficient	1.000
2.10 The number of pages is adequate	1.000
2.11 The size of the title and section headings is adequate	1.000
**Relevance**	
3.1 The educational manual will have an impact on healthcare delivery	1.000
3.2 The material encourages the healthcare team to change their approach to patient care in PC using hypodermoclysis	1.000
3.3 The information directed toward the object of interest is sufficient and appropriate	1.000
3.4 Suitable for use by any healthcare professional in their educational activities.	1.000

**Table 2:** Recommendations from content judges that were accepted.

Prototype	Suggestion from the Expert Judge	Decision
Internal Page Layout	JC3, JC8: Adjust pages where illustrations overlapped the content, hindering readability	Adjusted with the designer
Compatibility Table and Medication Lists	JC6: Remove Ranitidine from the list, in accordance with Resolution RE No. 3.259/2020	Removed from the manual
	JC6: Adjust the information regarding compatibility among tramadol, hyoscine, and dipyrone	Adjusted in the manual
	JC6: Adjust the information considering that, in Brazil, hyaluronidase is restricted to esthetic use	Adjusted in the manual
	JC6: Adjust the permitted volumes per puncture site as described	Adjusted in the manual
Symptom Management	JC13: Insert the Edmonton Scale in the symptom management chapter	Adjusted in the manual
Proposed Care Measures	JC6, JC8, JC14: Revise words and standard operating procedure guidelines, adjusting and replacing expressions to improve understanding of the care purpose	Adjusted in the manual

**Table 3:** Total SAM scores according to the evaluation of visual design judges.

Technical Judge	Content	Language	Illustrations	Presentation	Stimulation and Motivation	Cultural Appropriateness	SAM Total Score	Interpretation According to Percentage Score (%)
JD1	6	9	3	4	5	4	31	91%
JD2	6	9	3	3	6	4	31	91%
JD3	6	10	3	4	5	3	31	91%
JD4	5	10	3	3	5	4	30	90%
	23	38	12	14	21	15	123	
Total	96%	95%	75%	88%	88%	94%	96%	91%

## Data Availability

The raw data supporting the conclusions of this article will be made available by the authors on request.

## Supplementary Materials

The following supporting information can be downloaded at: https://www.mdpi.com/article/10.3390/nursrep15080301/s1, Table S1. Table of Contents: Hypodermoclysis Educational Manual.

## Public Involvement Statement

No public involvement in any aspect of this research.

## Guidelines and Standards Statement

This manuscript was drafted in accordance with the stages proposed by Oliveira, Lucena, and Echer [12].

## Abbreviations

The following abbreviations are used in this manuscript:

CINAHL	Cumulative Index to Nursing and Allied Health Literature
CVI	Content Validity Index
DECS	Descritores em Ciências da Saúde (Portuguese)
DHC	Descending Hierarchical Classification
LILACS	Latin American and Caribbean Health Sciences Literature
MeSH	Medical Subject Headings
PC	Palliative Care
PDF	Portable Document Format
SAM	Suitability Assessment of Materials
SOP	Standard Operating Procedures
