# Supplementary material for: Development and Validation of an Educational Tool on Hypodermoclysis for Palliative Care Professionals
Source: Nurs Rep. 2025 Aug 16;15(8):301. doi: 10.3390/nursrep15080301 (PMC12389239; doi:10.3390/nursrep15080301)
Supplement: Supplementary file 1 [file nursrep-15-00301-s001.zip › nursrep-3784100-supplementary.pdf]

**Supplementary Table S1 – Table of Contents: Hypodermoclysis Educational Manual**

| Section Title | Topics Covered |
|---|---|
| **Preface** | Introduces the context, objectives, and collaborators involved in the development of the manual, emphasizing the institutional background and rationale. |
| **Palliative care: history and meaning** | Outlines the global history of palliative care (PC), including core principles such as holistic and interdisciplinary approaches to end-of-life care. |
| **Symptom control** | Describes common symptoms in PC patients and nursing strategies for their assessment and management, including pharmacological and non-pharmacological interventions. |
| **Patient safety** | Covers foundational concepts of patient safety (PS), national policies, risk mitigation strategies, and the integration of safety culture in healthcare teams. |
| **Skin and lymphatic system** | Explains the anatomy and physiology of the skin and lymphatic system, highlighting their relevance to subcutaneous (SC) drug absorption and infusion. |
| **Hypodermoclysis** | Presents the technique of hypodermoclysis (HDC) as a viable method for symptom control in PC, including its historical development and clinical justification. |
| **Legislation and nursing council opinions** | Summarizes Brazilian legal regulations and official statements from nursing councils (e.g., COFEN – Federal Nursing Council of Brazil), which regulate the use of HDC by nursing professionals. |
| **Indications and contraindications** | Lists clinical conditions in which HDC is recommended (e.g., dysphagia, poor venous access), and scenarios where it is contraindicated. |
| **Advantages** | Details the clinical and logistical benefits of SC infusion, such as ease of use, comfort, low infection risk, and reduced hospitalization. |
| **Disadvantages** | Discusses limitations of the technique, including medication restrictions, local adverse effects, and fluid volume limitations. |
| **Subcutaneous and parenteral route comparison** | Compare SC, intravenous (IV), and intramuscular (IM) routes in terms of drug absorption, onset of action, and patient suitability. |
| **Pharmacokinetics and bioavailability** | Explains how medications behave when administered via SC route, including absorption rates, plasma levels, and clinical implications. |
| **Sites of administration** | Describes recommended anatomical sites for SC infusion in adults and pediatric patients, with practical guidance for volume and positioning. |
| **Contraindicated areas** | Identifies anatomical regions where SC infusion should be avoided due to poor absorption, risk of complications, or previous surgery. |
| **Complications and adverse events** | Lists potential local and systemic complications (e.g., edema, necrosis, allergic reactions) and outlines prevention and response strategies. |
| **Medication administration** | Provides instructions for drug preparation, dilution, and administration techniques for SC infusion, including bolus and continuous methods. |

| | |
|---|---|
| **Off-label medication uses in palliative care** | Addresses the ethical and clinical rationale for off-label use of medications in PC, supported by international literature. |
| **Medications used and compatibility table** | Includes a comprehensive list of medications compatible with SC infusion and their safe combinations based on Brazilian practice guidelines. |
| **Unviable medications for hypodermoclysis** | Highlights drugs that should not be used via HDC due to the high risk of tissue damage or lack of efficacy. |
| **Nursing care and puncture technique** | Details the technical steps for safe catheter insertion, including angle of entry, site selection, and maintenance. |
| **Standard Operating Procedure** | Presents the full Standard Operating Procedure (SOP) for performing HDC in clinical settings. |
| **Nursing care after a puncture** | Explains post-procedure monitoring, dressing care, site evaluation, and patient education to ensure safe and effective outcomes. |
| **References** | |
